# The effect of sexuality education based on the information, motivation, and behavioral skills model on improving the teachers’ professional competence

**DOI:** 10.1016/j.heliyon.2024.e24170

**Published:** 2024-01-07

**Authors:** Raziyeh Maasoumi, Seyed Ali Azin, Saharnaz Nedjat, Moslem Parto, Arshia Zamani Hajiabadi, Keshvar Samadaee Gelehkolaee

**Affiliations:** a Department of Reproductive Health, School of Nursing and Midwifery, Tehran University of Medical Sciences, Tehran, Iran; b Nursing and Midwifery Care Research Center, School of Nursing and Midwifery, Tehran University of Medical Sciences, Tehran, Iran; c Reproductive Biotechnology Research Center, Avicenna Research Institute, ACECR, Tehran, Iran; d Department of Epidemiology and Biostatistics, Tehran University of Medical Sciences, Tehran, Iran; e Faculty Member of Organization for Educational Research and Planning (OERP)-Research Institute for Education (RIE), Tehran, Iran; f Student research committee, School of Medicine, Mazandaran University of Medical Sciences, Sari, Iran; g Sexual and Reproductive Health Research Center, Department of Reproductive Health and Midwifery, Faculty of Nursing and Midwifery, Mazandaran University of Medical Sciences, Sari, Iran

**Keywords:** School-based sexuality education, "Teachers' professional competence", Adolescent, IMB model, Iran

## Abstract

**Introduction:**

Professional competence is the basic need of teachers in effective sexuality education. Therefore, the aim of this study was to evaluate the impact of school-based sexuality education (SBSE) on teachers' professional competence (TPC), using the information, motivation, and behavioral skills (IMB) model, in boys' schools.

**Methods:**

A randomized controlled field trial was conducted on 60 teachers who taught adolescents aged 11–19 years and were selected from 12 public boys' schools in Sari, northern Iran. Two groups (intervention and control) were assigned using a multi-stage stratified random sampling method. Researchers utilized a self-reported socio-demographic questionnaire and an IMB model-based questionnaire to assess the effects of the educational program. Four groups of 6–8 people underwent six 2-h training sessions based on an IBM model. Teachers were assessed before, immediately, and six weeks after the intervention to evaluate the outcome variables. The data were analyzed using the software SPSS-V19 and Chi-square test, Independent *t*-test, One-way ANOVA, and Repeated Measure ANOVA.

**Results:**

There were no significant differences between intervention and control groups at the baseline in socio-demographic characteristics and TPC (p > 0.05). The mean scores of TPC in sexuality education in every three dimensions of knowledge (P = 0.001), skill (P = 0.002), and attitude (P = 0.007) were significantly higher in the intervention group than in the control group.

**Conclusions:**

The results of this study show that by using the SBSE program based on the IMB model, the TPC for teaching sexual issues can be improved.

## Highlights

• Sexuality education requires sufficient knowledge and a positive attitude toward sexuality.
• Creating a creative environment helps students in learning skills.
• Teachers play a crucial role in creating creative thinking and responsible decision for students.
• Educational policymakers should pay more attention to the training of teachers to improve their professional competence in sexuality issues.

## Abbreviations

**IMB**: Information, motivation and behavioral skills
**TPC**: Teachers' professional competence
**SBSE**: School-based sexuality education
**SRH**: Sexual and reproductive health

## 1 Introduction

Adolescence is a period of personal development and self-improvement, a time of discovering and defining personal limits as part of the process of becoming an adult. Therefore, it can be called a vital opportunity to ensure a successful transition to adulthood [1,2]. The physical, psychological, and sexual changes that occur during this period create confusion for them. Due to a lack of awareness about these changes and deficiencies in providing services to adolescents, they are exposed to risks such as unwanted pregnancy, sexual violence, and sexually transmitted infections. These threaten their life and future [3]. On the other hand, the concept of masculinity creates unequal gender norms, roles, and power in the world. Therefore, gender can be considered a risk factor for health behavior [4]. One of these inequalities is the deficiency in sexual and reproductive health services for men [5]. The WHO recommended a gender-transformative approach that could partially reduce this inequality for men [6]. Therefore, it seems that the sexual socialization process of men needs more work.

Sexual socialization, the process of learning about sexuality, is shaped by various factors including family, education, friends, and society [7]. Teachers have a key role in this process [8]. During education, teachers impart not only specialized knowledge, but also their experiences and ideologies to their students [9]. Therefore, having knowledge, a positive attitude, and skills in teaching or teachers' professional competence (TPC) in the field of sexual issues can play an important role in promoting adolescent sexual health [10]. Due to the increasing awareness of adolescent rights in Iran, there is a growing demand for access to comprehensive sexual health services and information [11]. Also in the world, the types of interventions have been employed to increase adolescents' reproductive health [12]. School-based sexuality education (SBSE) interventions for adolescent sexual health are some of the notable interventions in this regard [1,12]. Age-appropriate educational content, cooperation with parents, and the use of TPC are the principles of an effective SBSE intervention [13,14]. TPC refers to the acquisition of knowledge and skills, and the motivation to activate learners' capacity to interact properly with the community around them [15]. The concept's conceptual framework is in line with the Information, Motivation, and Behavioral Skills (IMB) model. According to this model, several key factors affect the empowerment of teachers in the field of sexual education for adolescents. Based on this model, it appears that the empowerment of teachers in the realm of sexual education for adolescents is impacted by a few key factors. To effectively assist adolescents in managing their sexual behavior, it is important to possess knowledge of the high-risk behaviors they may engage in, be motivated to help them, and have the necessary skills to educate and address any questions they may have. Teachers can enhance sexual education by focusing on these elements and guiding informed decision-making. The model that Fisher et al. developed in 1996 have been shown to be incredibly effective at reducing the risky behaviors of adolescents. It's important to continue promoting the use and development of this approach, as it has made a real difference in the lives of young people. By providing effective strategies and support, we can empower young people to make informed decisions and lead healthy and fulfilling lives [16,17]. Extensive research has been conducted to determine the role that the environment and educational content, particularly schools, play in attaining a sound biological capacity. However, the proper implementation of appropriate educational content also requires extensive effort [18,19].

### 1.1 Adolescent's sexuality Education in Iran's Context

Iranian adolescents, especially boys, have numerous educational needs in the field of sexual health that have not been met due to different barriers [5]. They lack access to age-appropriate sexual and reproductive health services [20]. Health issues related to unwanted pregnancies, sexual harm, and sexually transmitted diseases require effective policymaking [[21], [22], [23], [24]]. One of the most common harm reduction strategies for adolescents is the educational approach [21]. However, there is a disagreement about adopting an educational approach to sexuality education [25]. In many conservative societies, such as Iran, the approach to sexual health education is often focused on abstinence-only. This approach seems to stem from a broader socio-cultural or political ideology rather than a practical consideration of existing sexual health conditions [11,26,27], While studies have confirmed the comprehensive sexuality education approach to having a safe life for adolescents [11,28] but for reasons such as teachers' professional incompetence, teachers' discomfort with the content of sexual education, and legal and cultural barriers, this educational approach is not applicable in Iranian society [5,29,30]. Therefore, designing interventions to improve teachers' professional competence can be an effective solution [14]. Different factors affect the effectiveness of interventions, including interventions based on health promotion models [31]. Double gender standards, especially regarding adolescent sexual behavior and gender confirmation, are risk factors for high-risk behaviors [32,33]. Therefore, the purpose of this study was to assess the effect of school-based sexuality education (SBSE) based on the IMB skills model on improving the teachers’ professional competence (TPC) in boys' schools.

## 2 Methods

### 2.1 Research design

The present study was a randomized controlled field trial that has been carried out on teachers of boys' first and second secondary schools (adolescents aged 11–19 years). This project is the third phase of the Ph.D. thesis of the corresponding author. After registering the project in the ClinicalTrials.gov *PRS* with ClinicalTrials.gov ID: NCT04275271 and receiving the necessary permits to submit to the Department of Education of Sari, Sari is a city in the north of Iran, comprising 2 municipal districts with a population of 350,000 people and 32 public boys' schools (First and second high schools). All participants were informed about the purposes of the study, voluntary participation, confidentiality of information, and evaluation methods. After receiving written consent from the participants, between May to August 2020, the interventional program was presented by the corresponding author (after undergoing a comprehensive sexuality education course under the supervision of a team sent to Iran by UNFPA).

### 2.2 Population and sampling

The sample size was calculated at 30 persons in each group based on the mean difference scores between two groups with 95 % confidence level, 80 % power, and considering 20 % attrition rate [34].

**Fig. 1:**
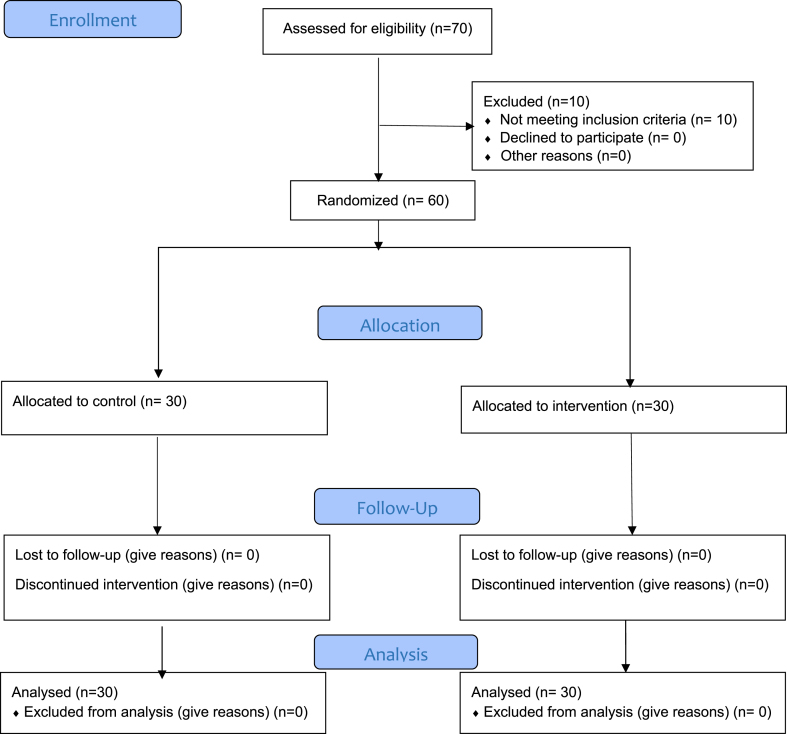
Flow diagram of the participant.

12 all-boys schools were randomly selected from the 32 public high schools that were located in Sari (municipal districts 2) with a multi-stage stratified random sampling method (Fig. 1). An invitation was sent to school principals and the schools were randomized to control and intervention groups. In the next step, the volunteer teachers were selected randomly from the teacher's list of each school and they were informed about the objects of study by the WhatsApp group. The enrollment and data collection process was about a four-month period, from May to August 2020.

The inclusion criteria include "willingness to participate in the study, having at least 5 years of teaching experience in boys' schools, teaching adolescent boys aged 11–19, having general health due to coronavirus disease (due to the coincidence of the beginning of classes with the coronavirus pandemic)". The exclusion criteria include "absence in one-third of sessions, manifestations of coronavirus disease, and participating in previous sexuality education courses.

### 2.3 Program

Educational interventions were designed in the form of 4 groups of 6–8 people and six 2-h sessions that were delivered two per week (Table 1). Two pre-trained male facilitators (medical students) assisted in the pre-test, post-test, and teaching exercises during the sessions. The educational content was designed based on the sexuality education program (results of phase one of the study) [29] and localized by qualitative method (results of phase two of the study). In this phase, the data were collected through interviews using targeted questions based on the IMB skills model constructs and educational content was design [11]. This model is built on three key components: information, motivation, and behavioral skills. Information refers to all the knowledge and awareness about a particular phenomenon, including cognitive myths that can impact decision-making. Motivation encompasses personal beliefs, attitudes towards a behavior, and social factors such as perceived social support or social norms. Finally, behavioral skills include both objective skills and self-efficacy [31]. After approved the educational content by the research team, it was reviewed by four faculty members who were not part of the team. These reviewers had previously audited the project's earlier phases, and their input helped to ensure the content's validity. It is important to note that Iran currently does not have an official sexuality education course for teachers. However, in-service courses are recognized as life skills within the education system. After receiving the final correction in each session, the participants obtained the educational content through PowerPoint and a copy of the scenarios. The class continued with questions and answers, group discussions, and brainstorming. At the end of the training section, special exercises were presented based on the relevant topic for the class and homework. Two separate groups were created in WhatsApp (by gender) to respond to questions. The control group did not receive any training from the research team during the study. Slides and educational content were given to the control group after the follow-up. Another WhatsApp group was also formed for them to ask their questions. Fidelity to the executive protocol was monitored by the supervisor (first author) and an inspector from the provincial education department. Also, the Ministry of Health evaluation form was filled out by participants. Fidelity to the executive protocol was assessed with the following objectives: Were all the participants present? Were the discussions based on the assigned topics? Were health and social distance protocols in place to protect participants' health? Was the discussion participatory with the active presence of the participants? Were the assignments properly reviewed and given feedback?

**Table 1:** Content topics of intervention sessions.

Session	Key concepts	Topic	Description
First	Values, beliefs, rights, culture and gender	Participatory methods, how to evaluate, human rights, Sexual and reproductive rights, Religion, Sexuality and culture	Group discussion on human rights, examples of violations around, preparing a list of laws needed to create a suitable environment for sexuality education, Write examples that show gender and sex.
Second	Understanding gender and self-care	Gender norms and stereotypes, Gender-based violence, Privacy and media literacy, masculinity and outcomes.	Name a few examples of gender stereotypes, discuss its effects on health, list laws that reinforce gender-based violence, and discuss with each group ways to reduce violence according to existing laws. Play a role in defending human rights in defense of gender equality
Third	Sexuality and interpersonal relationships	Sexual behavior, sexual response cycle, family, responsibility, and romantic commitments and relationships	Each group expresses an experience of students' romantic relationships, discusses generational change, questions and answers about the organs involved in the sexual response cycle.
Fourth	Skills needed for health and well-being	Negotiation skills, Decision making, Seek help and support, Crisis Management. Self-awareness.	Prepare a report of real experiences of dealing with the crisis and how to manage it, plan a problem and draw a tree of problems and design solutions with Socratic questions and answers. Fill in the sexual desire worksheet.
Fifth	Human growth and development	Anatomy and physiology of the reproductive and sexual systems, Puberty and fertility, Self-image	Create thoughts with questions about puberty changes and how to manage them, express your feelings during puberty,
Sixth	Sexual and reproductive health	Pregnancy, Contraception, Sexually transmitted diseases (care, treatment, support)	Presenting about sexually transmitted diseases, playing cards of contraceptive methods. How to support people with AIDS, and men's participation in family planning programs

### 2.4 Data collection

The data collection tools included a socio-demographic characteristics questionnaire and a researcher-made questionnaire to assess TPC. The socio-demographic characteristics questionnaire includes age, gender, marital status, number and age of children, socio-economic status, the field of education, occupation, teaching level, and work experience. The primary outcome of this study was a change in the mean score of teachers' professional competence, which was measured in three dimensions of knowledge, attitudes, and skills and collected by a researcher-made questionnaire based on previous phases of the project. Data were collected at three-time points before the intervention, immediately after the intervention, and six weeks after the intervention. This questionnaire includes 50 items 17 items in the field of knowledge, 16 items in the field of skills, and 17 items in the field of attitude. Under the title of “teachers' professional competence (TPC),” the questionnaire was handed out to 10 specialists to determine the content validity ratio (CVR) and content validity index (CVI). The face validity of the questionnaire was approved by 10 teachers as lay experts. The questionnaire was given to 50 teachers to check the reliability and was filled out again after two weeks. The results showed ICC (knowledge domain 0.99, skill domain 0.99, and attitude domain 0.98) and Cronbach's alpha (knowledge domain 0.90, skill domain 0.89, and attitude domain 0.76) show a high level of reliability. The instrument scores using the Likert scale are 5 points [[1], [2], [3], [4], [5]]. A knowledge range of 1 means I don't know at all and a score of 5 means I know one hundred percent, skill range of 1 means I can't at all and a score of 5 means I can one hundred percent, and attitude Range Score 1 strongly disagree to 5 strongly agree. 3 items 27, 38, and 42 inverted points were assigned. Finally, each domain is calculated based on the sum of the scores of the same domain (knowledge, skills, and attitude). And higher scores indicate a better position for the participant in that area.

The data were analyzed using the software (SPSS–V19). The following statistical tests were used: the Chi-square test to compare categorical variables, the Independent Samples *t*-test to compare two sample means from unrelated groups, the One-way Analysis of Variance (ANOVA) to compare the means of two or more independent groups and determine whether there is statistical evidence that the associated population means are significantly different, and the Repeated Measures ANOVA to compare means across one or more variables based on repeated observations. All statistical calculations were performed at a significant level of (P < 0.05).

## 3 Results

In the present study, 70 people were initially included in the study. 10 people were excluded from the study due to not meeting the inclusion criteria. Finally, 60 people were randomly assigned into two intervention and control groups. The consort flow diagram shown in (Fig. 1). Results of the independent *t*-test, Fisher's exact test, and chi2 of the two groups were homogenous in terms of socio-demographic variables (P > 0.05) (Table 2). The mean age of the participants in the intervention and control groups was, respectively, 40.83 ± 8.39 and 39.83 ± 8.26 years. The majority of participants in the two groups were men 56.7 %. (Table 2).

**Table 2:** Demographic characteristics of participants.

Characteristics	Intervention group N = 30	Control group N = 30	*P* value
Age (years), Mean (SD)	40.83(8.39)	39.83(8.26)	0.64
Gender, n (%) Female Male	13(43.3) 17(56.7)	13(43.3) 17(56.7)	0.60
Education, n (%) Bachelor Master Degree PhD	15(50) 13(43.3) 2(6.7)	14(46.7) 14(46.7) 2(6.6)	0.96
Job, n (%)			0.48
Teacher	20(66.7)	16(53.3)	
Counselor	8(26.7)	8(26.7)	
Principal	1(3.3)	3(10)	
Manciple	1(3.3)	3(10)	
Socio-economic status Good Moderate Poor	11(36.7) 17(56.7) 2(6.6)	9(30) 18(60) 3(10)	0.80
Work experience (Years)			0.54
5–9	7(23.3)	5(16.7)	
10–14	5(16.7)	5(16.7)	
15–19	7(23.3)	12(40)	
>19	11(36.7)	8(28.7)	
Teaching level			0.67
First High School	10(33.3)	8(26.7)	
Secondary High school	13(43.3)	12(40)	
Both	7(23.4)	10(33.3)	
Field of education			0.74
Psychologist	11(36.7)	14(46.7)	
Sociologist	3(10)	3(10)	
Biologist	2(6.7)	3(10)	
Others	14(46.7)	10(33.3)	

Independent *t*-test on TPC in each domain (knowledge, attitude, skills) showed that there was no significant difference in the baseline between the two groups (p > 0.05) (Table 2). A one-way ANOVA test was used to investigate the relationship between socio-demographic variables and professional competence scores. There was a correlation between the field of education and the domains of TPC. The Tukey test was shown psychologist teachers in the knowledge domain (P = 0.001) and skill domain (P = 0.002), also sociologist teachers in the attitude domain (P = 0.007) obtained higher mean scores than the control group. (Table 3).

**Table 3:** Correlation between the Field of education and the TPC score according to the dimensions.

Outcome	Psychologist (n = 25)	Sociologist (n = 6)	Biologist (n = 5)	Others (n = 24)	ANOVA P-Value^a^
	Mean (SD)	Mean (SD)	Mean (SD)	Mean (SD)	
Knowledge	48.76(5.96)	46.81(8.94)	48.23(4.26)	38.66(6.26)	0.001
Attitude	89.35(6.31)	92.64(8.10)	79.41(4.41)	88.17(6.20)	0.007
Skills	57.81(8.31)	54.42(9.23)	50.31(6.28)	47.46(10.10)	0.002

a Ona-way ANOVA, P-Value<0.05 Significant. TPC: Teachers' professional competenc

**Fig. 2:**
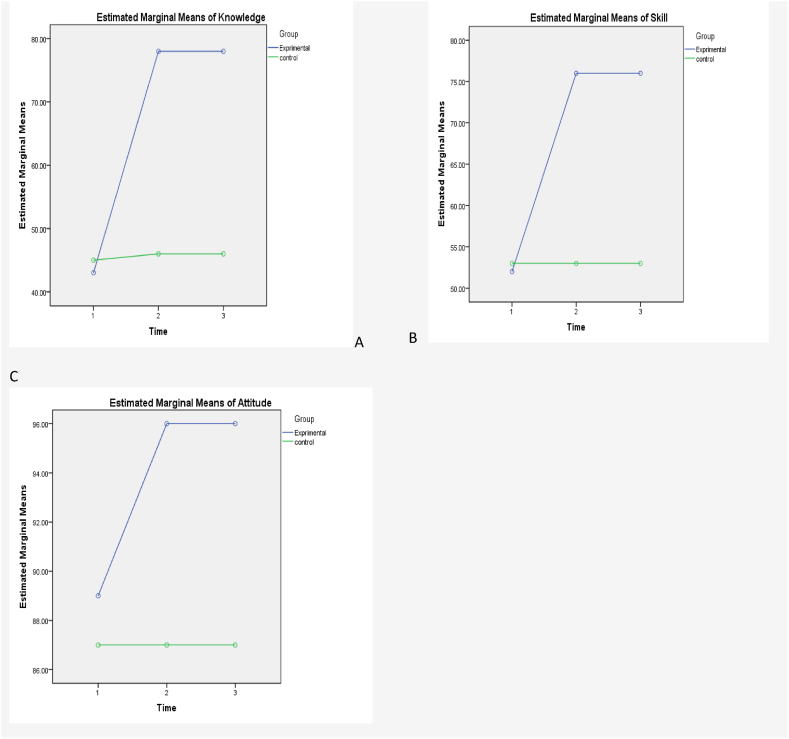
The comparison of the mean scores of teachers' professional competence in the knowledge (A), Skills (B) and attitude (C) dimensions in the two groups of intervention and control. (Before the intervention, immediately, and 6 weeks after the intervention).

The results of the Repeated Measure ANOVA in the intervention group showed increased mean scores in three areas of knowledge, skill, and attitude in a period of time. (Immediately after the intervention) (Table 4). Also, the changes in the mean scores of the three domains in the two intervention and control groups before, immediately after, and six weeks after the intervention is shown in the diagrams related to each area (Fig. 2).

**Table 4:** Descriptive and General Linear Model Analyses of three stages in intervention and control group and interaction between time and group.

Outcome	Pre-assessment I (n = 30) C(n = 30)		Post-assessments I (n = 30) C(n = 30)		Six-weeks follow-up I (n = 30) C(n = 30)	Time Within(I) P-Value^a^	Time Within(c) P-Value^a^	Time& group P-Value^a^
	Mean (SD)	Mean (SD)	Mean (SD)	Mean (SD)	Mean Mean (SD) (SD)			
Knowledge	43.38(7.45)	45.58(8.14)	78.38(6.30)	46.71(7.60)	78.48(6.29) 46.86(7.50)	0.001	0.290	0.24
Attitude	89.26(7.24)	87.50(6.47)	96.56(2.42)	87.40(6.50)	96.56(2.48) 87.40(6.47)	0.001	0.482	**1**
Skills	52.34(11.44)	53.07(8.60)	76.40(7.01)	53.90(8.90)	76.40(6.90) 53.95(9.07)	0.001	0.400	**1**

I = Intervention group.

C = Control group.

a Based on repeated measures ANOVA.

## 4 Discussion

The present study was a randomized controlled field trial aiming to enhance the TPC with culture-based sexuality education based on the IMB model. The results of the study showed that this educational model enhanced the TPC in all three dimensions of knowledge, skills, and attitudes. In the present study, the greatest change in the average knowledge and skill dimensions and the least change in the average score of attitude occurred before and immediately after the intervention. It is noticeable that before the intervention, the mean score of teachers' attitudes was higher, which indicates in the current Iranian society, teachers' attitudes toward the implementation of SBSE have become more positive, according to the socio-cultural conditions. Therefore, there are suitable conditions for it. While the teachers' lower scores in the other two dimensions show the need for skill-based educational interventions. These results confirm the results of the previous two phases of this project [11,35]. A positive attitude of teachers towards SBSE can contribute to more effective implementation and thus the possibility of reducing the risk of sexually transmitted diseases and unwanted pregnancy in adolescents [36].

Contrary to our finding, another study reported that the educational intervention was not effective in the dimension of knowledge but was effective in the dimensions of skill and attitude [37]. This difference could be due to the difference in the educational content and measuring tools of the two studies or the level of prior knowledge of the target group. Studies in line with the present study confirmed the effect of school-based interventions on all three dimensions [38,39]. In the present study, in accordance with Ahari's study, no change was observed in the mean scores of TPC dimensions after 6 weeks [40]. This can show the need to continue this type of education during life.

According to a study done by Suwarni et al., the IMB model made a significant difference in all three dimensions before and after the intervention [17]. Also, Bahrami et al. in Iran confirmed the effectiveness of interventions based on this model [16]. Therefore, this model can be recommended as an effective model for implementing SBSE in preventive and risk-reduction programs for adolescents.

In the present study, teachers with psychological education gained higher knowledge and skills in sexuality education than other teachers. While another study, school health teachers were more comfortable teaching the comprehensive sexuality education curriculum. In this study, teachers' self-efficacy and teachers' sense of comfort with the educational theme predicted teachers' commitment to teaching [41]. Differences in the educational system of different countries can cause this difference in results. However, this can be said that teachers' familiarity with sexual issues can play a role in motivating them to acquire teaching skills. The results of the present study were consistent with the results of a study in the Netherlands that stated teachers' understanding and knowledge of their skills are important factors in gender education [39]. Another study in Zimbabwe emphasized that teachers who work as counselors in schools should be trained and involved in sexuality education [19].

This study was in fact complementary to previous studies that had examined the effect of SBSE programs on based the IMB model for the promotion of TPC. Among the strengths of the study is its innovative goal, i.e., assessment of TPC in SBSE. Furthermore, the study has been designed using the IMB model, with the goal of guiding teachers toward behavioral change and changing their professional competence. Also, a questionnaire was designed consisting of 50 items to better assess their TPC. Teaching the protocol approved by individually trained specialists was one of the strengths of the present study. In this study, blinding was not possible due to the intervention by the researcher in this dimension, but the analyzer did not know about the intervention. In addition to ethical issues in human research, due to the pandemic of the Covid-19, all hygiene protocols were strictly followed. Restrictions on implementing training sessions due to the pandemic COVID-19 and the impossibility of implementing training classes for the control group were among the limitations of the present study. However, efforts were made to reduce the restrictions by providing incentives such as providing educational materials after the follow-up period for the control group and giving the educational certificate to the participating teachers. Finally, we recommend longitudinal research to assess this objective for future studies.

## 5 Conclusion

The SBSE intervention based on the IMB model could be enhanced the TPC in boys' schools. It is notable that policymakers, school principals, and families support these programs.

## Funding

This study has not been funded.

## Availability of data and materials

The datasets used and/or analyzed during the current study available from the corresponding author on reasonable request.

## Ethics approval and consent to participate

The study was part of a Ph.D. Thesis supported by the Tehran University of Medical Sciences with Ethic Code (IR.TUMS.FNM.REC.1397.102). Informed consent was received from all participants. Participation in the survey was voluntary as participants could decline to participate at any time during the study.

## Consent to participate

Informed consent was obtained from all individual participants included in this study.

## CRediT authorship contribution statement

**Raziyeh Maasoumi:** Writing – review & editing, Supervision, Conceptualization. **Seyed Ali Azin:** Writing – review & editing, Supervision, Conceptualization. **Saharnaz Nedjat:** Supervision, Methodology, Formal analysis. **Moslem Parto:** Writing – review & editing, Conceptualization. **Arshia Zamani Hajiabadi:** Writing – original draft, Data curation. **Keshvar Samadaee Gelehkolaee:** Writing – review & editing, Writing – original draft, Validation, Methodology, Conceptualization.

## Declaration of competing interest

The authors declare that they have no known competing financial interests or personal relationships that could have appeared to influence the work reported in this paper.
